# Announcement

**Published:** 1891-01

**Authors:** 


### ANNOUNCEMENT.

It is with great pleasure that we present THE JOURNAL to our readers, with this issue, free from the objectionable insert advertising pages which have so long marred its beauty. It has been the intention of the present owners of THE JOURNAL ever since its purchase to leave them out, but owing to existing contracts we have been unable to do so before.

The success of THE JOURNAL during the last year has far surpassed any previous record. Our subscription list is rapidly growing, and every mail brings us new subscribers and encouraging letters of appreciation. During the year 1890 we have registered on our books new subscribers from almost every State and territory in the Union. Our *bona fide* circulation for 1890 was more than double that of 1889, and we hope to greatly increase it during 1891.

This issue will go to many who are neither subscribers nor contributors to THE JOURNAL. We want you to read our JOURNAL. We want you to write for it. We want you to report your interesting cases, that you and the profession at large may be benefited thereby.

THE JOURNAL will always advocate with zeal that which is highest and best in the profession, and condemn without fear that which tends to lower its tone or usefulness. There are measures of especial importance that will be brought before the adjourned session of the legislature, which will be outlined and vigorously advocated in future issues. The laws regulating the practice of medicine in Georgia need to be amended, and existing laws should be more rigidly enforced.

